# Significance of Urinary Proteome Pattern in Renal Allograft Recipients

**DOI:** 10.1155/2014/139361

**Published:** 2014-03-13

**Authors:** Sufi M. Suhail

**Affiliations:** ^1^DUKE-NUS Graduate Medical School, Singapore 169857; ^2^Department of Renal Medicine, Singapore General Hospital, 20 College Road, Singapore 169856

## Abstract

Urinary proteomics is developing as a platform of urinary biomarkers of immense potential in recent years. The definition of urinary proteome in the context of renal allograft and characterization of different proteome patterns in various graft dysfunctions have led to the development of a distinct science of this noninvasive tool. Substantial numbers of studies have shown that different renal allograft disease states, both acute and chronic, could portray unique urinary proteome pattern enabling early diagnosis of graft dysfunction and proper manipulation of immunosuppressive strategy that could impact graft prognosis. The methodology of the urinary proteome is nonetheless not more complex than that of other sophisticated assays of conventional urinary protein analysis. Moreover, the need for a centralized database is also felt by the researchers as more and more studies have been presenting their results from different corners and as systems of organizing these newly emerging data being developed at international and national levels. In this context concept of urinary proteomics in renal allograft recipients would be of significant importance in clinical transplantation.

## 1. Introduction

Overt proteinuria is an established prognostic marker in renal allograft recipients associated with allograft dysfunction and graft loss [1, 2]. However, the early detection of the causes of graft dysfunction and graft loss is important. The current modality for definitive diagnosis of graft abnormalities is graft biopsy [3]. Inherent risk of biopsy on a single transplanted kidney and delay involved in the detailed reporting of the tissue could preclude an early diagnosis of the graft dysfunction and early institution of specific therapy. On the other hand, urinary proteome is a distinct entity from the conventional nosology of proteinuria that has been emerging in recent years [4, 5]. Urinary proteome constitutes the entire genomic protein content that is excreted in urine in health and disease states. Proteomic urine analysis could predict the diagnosis of renal transplant pathologies early, which could impact the graft function and survival in the long run [5]. Moreover, urinary proteome patterns in transplant patients could differentiate stable graft function from acute tubulointerstitial rejection (AR), urinary tract infection (UTI), acute tubular necrosis (ATN), and calcineurin inhibitor (CNI) toxicity [6]. In addition, characterization of chronic allograft dysfunction into chronic antibody associated rejection (CAAR), interstitial fibrosis tubular atrophy without inflammation (IFTA), chronic recurrent or de-novo glomerulonephritis (GN), and transplant glomerulopathy (TG) could also be predicted by urinary proteome pattern [7, 8].

Over the current decade, several proteome data sources have revealed a large pool of discovery and sequencing of previously unexplored urinary peptides and protein chains in health and disease states [9]. Furthermore, system for organizing the data structure of several proteome has also been generated through hierarchical tree that yields high-quality protein families which come from various databanks, that is, GenBank, Protein Data Bank (PDB), SwissProt, Protein Information Resource (PIR), and Protein Research Foundation (PRF) [10]. The aim of this general review is to elucidate the aspects of urinary proteome patterns in different renal allograft pathologies.

## 2. Overt Proteinuria in Renal Allograft Recipient

While pretransplant proteinuria from the native kidney disappears rapidly after renal transplantation [11], persistence of overt proteinuria (urine protein creatinine ratio > 0.2 g/g) in renal transplant recipients implies glomerulonephritis (GN), transplant glomerulopathy (TG), and mammalian target of rapamycin (mTOR) induced graft nephropathy. This is strongly associated with poor graft survival [1, 12]. In chronic kidney disease (CKD), this overt proteinuria could be stratified by qualitative analysis into low molecular weight (20,000 Da to 33,000 Da) tubular protein and middle and high molecular weight (60,000 Da to 150,000 Da) glomerular protein. This stratification enables identification of their association with different types of glomerular and tubular lesions in CKD (Table 1) [13, 14]. However, similar stratification in renal allograft showed that low molecular weight tubular protein could be present despite good graft function [14], while glomerular proteinuria poses a statistically significant adverse outcome on graft function and survival [2]. Allograft biopsy in transplant glomerulopathy with chronic graft dysfunction (CGD) revealed glomerular abnormalities leading to glomerular type of proteinuria [15]. In one study positive correlation between glomerular proteins and inflammatory cytokines in renal tissue was found in patients with poor graft function [16].

On the other hand, allograft dysfunctions related to acute tubulointerstitial rejection (AR), CNI toxicity, acute tubular necrosis (ATN), interstitial nephritis (IN), urinary tract infections (UTI), chronic antibody associated rejection (CAAR), and interstitial nephritis tubular atrophy (IFTA) are not associated with overt proteinuria. Even though these conditions do not induce significant proteinuria, they are associated with excretion of genomic proteins, known as urinary proteome, that might remain undetected by usual clinical laboratory methods for proteinuria. Nevertheless, these conditions imply major graft pathologies requiring precise and early detection, as delay in management could herald chronic and relentless course of graft loss.

## 3. Urinary Proteome in Renal Allograft Recipient

In contrast to overt proteinuria, smaller genomic peptides and protein chains ranging from 1000 Da to 20,000 Da present in urine are not detected by usual biochemical and immunological test for urinary protein in the clinical laboratory [4, 17]. Moreover, these urinary proteomes could be 1000-fold lower in concentration than that present in plasma and require liquid chromatography (LC) or capillary/column electrophoresis (CE) for separation, mass spectrometry (MS) for detection, and time-of-flight (TOF) measurement for pattern analysis [18].

These urinary proteomes display specific patterns in TOF-MS assay depending on the disease characteristics (Table 2). Urine, collected noninvasively and preserved and prepared with specified standards and protocols, is used in small volume in microliter (*μ*L) to identify and characterize large number of peptides and protein chains [4]. Over the current decade, these methodologies resulted in a large pool of discovery and sequencing of previously unexplored proteome in health and various pathologic states as can be explored from various proteome data sources, that is, GenBank, Protein Data Bank (PDB), SwissProt, Protein Information Resource (PIR), Protein Research Foundation (PRF), and so forth [19, 20].

## 4. Identification of Urinary Proteome

Techniques for urinary proteome analysis are of two types, gel-based (2-DE and 2D-DIGE) and gel-free (MALDI, SALDI, and iTRAQ) [18, 21, 22]. The gel-based detection of biomarkers is the first dimension isoelectric focusing, separating proteins in electrophoresis strips based on charge selectivity, and the two-dimensional gel electrophoresis (2-DE) which is the most widely used approach [21]. Sodium dodecyl sulfate polyacrylamide gel (SDSPAGE) electrophoresis is one common type of 2-DE that separates proteins on molecular size selectivity [13]. A more sensitive and high-throughput gel-based technique termed as two-dimensional difference gel electrophoresis (2D-DIGE) has developed in recent years [23]. These gel-based methods are used to identify low, middle, and high molecular weight proteins in urine.

The gel-free method is used to identify the smaller urinary proteins that consist of genomic urinary proteomes present in urine in small quantity [18]. The labeled identification of numerous proteomes using appropriate probe or antibodies is virtually impossible in the general perspectives. Therefore, label-free identification of proteomes of urine using direct liquid chromatography-tandem mass spectrometry (LC-MS) and column/capillary electrophoresis mass spectrometry technique (CE-MS) is used for separation and characterization in terms of their mass and charge* (m/z)* characteristics [7, 21, 24]. These methods are based on non-gel-based capillary or column electrophoresis using matrix assisted laser desorption-ionization (MALDI) or surface enhanced laser desorption-ionization (SALDI) of peptides placed on protein chips [7, 8]. Small aliquot of urine in microliter (*μ*L) is placed on these protein chips for capillary or column electrophoresis. The proteins are identified by their migration characteristics that depend on their molecular size/charge* (m/z)* and time-of-flight (TOF) characteristics. The whole process is abbreviated as CE coupled with MALDI-TOF-MS or SELDI-TOF-MS [7, 8]. Another very sensitive gel-free technique known as isobaric tags for relative and absolute quantification (iTRAQ) is also a method of choice [22].

As the detailed description of these techniques is beyond the scope of this clinical paper, we have summarized their salient and pertinent features in Table 3. The details have been described elsewhere [1, 7, 13, 18, 21, 23, 24].

## 5. Baseline Sources of Urinary Proteome

All constitutional renal cells ranging from glomerular podocytes to interstitial fibroblasts could be the sources of baseline urinary proteome. In renal allograft, additional immune-modulating cells that populate the grafted kidney because of host-graft interaction also could be a source of these proteins. Furthermore, selective upregulation and downregulation of these cells add to the additional novel proteins in urine [19]. Proteins released from these cells are recycled by tissue macrophages and tubular epithelial cells. The remaining substances are spilled over to urine and usually do not exceed 0.2 g/g of creatinine [4]. Therefore, proteomes are present in urine in the absence of overt proteinuria. Human Kidney and Urine Proteome Project (HKUPP.org) has set up a urine proteome pool of glomerular and tubular origin for a control database [20].

## 6. Baseline Proteome Pattern in Renal Transplant

The initial baseline urine proteome pattern remains fairly stable at different times of stable renal function of an individual, whether transplant or nontransplant. This was demonstrated in normal individuals at different intervals, in kidney donors before and after nephrectomy, and in renal transplant recipient's immediate posttransplant surgery and at later dates of stable graft function [25]. As transplantation is an altered state of homeostasis because of host-graft interaction, the proteome pattern shows unusual components in the mass spectrometry profile even with normal graft function [25]. However, unusual components at certain molecular weight of 3370, 3441, 3385 (human defensin), 4303, 10350, and 11732 Da (beta-2-microglobulin) are present in stable renal transplant patients [25]. Over the time, these proteome patterns remain fairly uniform as long as the graft function remains stable [25, 26]. Contrary to it, different graft dysfunctions could be associated with variations in urinary proteome pattern with the upregulation and downregulation of disease specific cells in renal tissue [26, 27]. This important aspect of proteome analysis indicates that urinary proteome could be a dependable biomarker for the prediction of different graft dysfunctions ahead of histopathological confirmation of graft pathology.

## 7. Urinary Proteome Patterns in Allograft Dysfunctions

### 7.1. Acute Allograft Rejection (AR)

In acute renal allograft rejection, upregulation of major histocompatibility cell (MHC) associated antigens led to the activation of antigen presenting cells (APC), B and T lymphocytes, natural killer cells (NK), tissue macrophages, and other inflammatory cells. These lead to the increased production of adhesion molecules, cytokines, perforin, and granzyme, as well as activation of complement cascade and coagulation cascade proteins, releasing their peptides and protein degradation products [6]. These peptides with several other molecules from apoptotic and necrotic cells produce spectral pattern of urinary proteome that have distinct spikes with higher signal intensity in the capillary or column electrophoresis mass spectrometry (CE-MS) [6, 8]. As shown in animal model of acute rejection in rat, proteome analysis with MALDI-TOF-MS platform revealed differential polypeptides as compared to normal control [27]. In addition, urine proteome analysis by SELDI-TOF-MS platform in allograft acute rejection revealed peptides that included mass (molecular weight) of 2003, 2807, 4756, 5872, 6990, 19018, and 25665 Da with sensitivity of 90.5% and specificity of 77.8% [28].

### 7.2. Acute Tubular Necrosis (ATN)

Proteome analysis with SELDI-TOF-MS of urine of renal allograft with acute tubular necrosis (ATN) consistently showed spectral patterns of polypeptide biomarkers with molecular weight bands of 6300, 28500, 33000, 43000, and 66000 Da in contrast to lower molecular weight peptides of acute rejection [29]. These small proteins identified in ATN are neutrophil-gelatinase-associated-ligand (NGAL), kidney-injury-molecule-1 (KIM-1), sodium-transporter-at-apical-membrane (NaHE-3), and so forth. Tubular biomarkers like adenosine-deaminase and carbonic-anhydrase-enzyme and spill over proteins like retinol-binding-protein and alpha-1-microglobulin, and so forth, were also included in this group of proteome [30].

### 7.3. Calcineurin Inhibitor (CNI) Toxicity

Calcineurin inhibitors (CNI) have long been known to have toxic effect on renal allograft. Experiment with cultured human embryonic kidney cells exposed to cyclosporine and tacrolimus showed proteomic perturbation, both intracellular and extracellular, in capillary electrophoresis. Several peptides and small proteins were identified as a result of upregulation of cyclophilin and downregulation of fibronectin [31]. Changes in macrophage activating factor were also identified [31]. In murine model, specific peptides originating from tubular cellular toxicity were also identified [32]. These peptides could be used as potential biomarkers for CNI toxicity.

### 7.4. Chronic Allograft Dysfunction

Chronic allograft dysfunction is characterized by chronic antibody associated rejection with C4d deposits (CAAR) and interstitial nephritis tubular atrophy (IFTA) most commonly [33]. The other causes of interstitial fibrosis are recurrent glomerulonephritis and transplant glomerulopathy. In these conditions, glomerular podocytes and tubular epithelial cells undergo mesenchymal transformation to form myofibroblast cells in the renal interstitial tissue, known as epithelial-to-mesenchymal-transformation (EMT) [34]. Myofibroblast cells derived from EMT lead to interstitial fibrosis in chronic allograft dysfunction. Renal allograft which showed markers of EMT on their podocytes and tubular epithelial cells in higher proportions developed diffuse interstitial fibrosis (IFTA) more frequently at five years after transplant [34]. Paxillin containing adhesion molecules and alpha-smooth muscle actin expression on glomerular podocytes and tubular epithelial cells were upregulated in these groups of transplant patients [34].

Study using label-free identification of urinary proteomes to characterize proteome pattern in CAAR and IFTA revealed significant differences in proteome patterns as compared to that of healthy individuals and renal transplant patients with stable graft function [7]. Using MALDI-TOF mass spectrometry the authors have shown comparative bioinformatics differences in urinary proteome pattern in CAAR, IFTA, stable transplant patients, and healthy individuals [7]. In all cases allergic drug reaction, sepsis, and other inflammatory process leading to graft dysfunction had been clinically excluded. In other studies, proteomes were mapped and characterized by genome wide expression profile in tissues [33]. Multiple sets of proteome with unique expression profile differentiating normal transplant biopsy and biopsies with IFTA and CAAR were mapped and validated, differentiating normal transplant biopsy from the biopsies with IFTA and CAAR [33].

## 8. Biomarker Data

Several works on renal allograft have identified different proteome patterns of urine in acute rejection, acute tubular necrosis, calcineurin toxicity, urinary tract infection, interstitial fibrosis tubular atrophy, and chronic antibody associated rejection. Moreover, studies demonstrated that these proteome patterns of various posttransplant states are distinct from the proteome patterns of the stable transplant recipients and healthy individuals [6–8]. Similarly, differences in urinary proteome patterns have also been demonstrated in native kidney glomerulonephritis [35, 36]. Currently, human urinary proteome project (HKUPP.org) has been updating the proteome pattern of human glomerulus and tubule in normal subjects and proteinuric subjects [20].

## 9. Limitations in Urinary Proteome Analysis

The major limitation in proteomic analysis of urine is the collection, preservation, and preparation of the urine sample, as urine is a biological product which tends to lose the diagnostic biomarkers over time due to degradation by its own protease enzymes and acquired bacterial contaminants. These can be obviated by following standard protocols for sample collection, storage, and preparation, as well as by using standard analytical performances of reliable 2DE, LC-MS, and CE-MS protocol described elsewhere [4, 13, 18]. Even though mandatory standards are followed, problems are encountered, particularly with the reproducibility of proteome patterns and noncomparability of data from different groups with divergent approaches. However, systems for organizing the data structure of several proteome pools have also been generated. Various databanks, that is, GenBank, Protein Data Bank (PDB), SwissProt, Protein Information Resource (PIR) and Protein Research Foundation (PRF), and so forth, have proposed unique hierarchical tree that yields high-quality proteome families that could bring harmony in data pool for disease prediction [10]. Last but not least, appropriate and user-friendly bioinformatics software for data evaluation, together with a formatted data bank as mentioned above, could eliminate the hindrance toward a globally approached and universally accepted system of urinary proteome analysis that could bring uniformity in disease prediction in this regard [37].

Hence a centralized proteome database could be of value for clinical application. A data sharing platform for data collection and integration from all urinary proteome projects could help to unify the urinary proteomes for collective use.

## 10. Conclusions

The voided urine of renal allograft recipient contains proteomes that have multiple sources based on allograft pathology. Consequently, the urinary proteome pattern analysis might differentiate the various graft abnormalities at an early stage of graft dysfunction. Conventional investigations including transplant biopsy to confirm the diagnosis require time and involve risk. Furthermore, delay in the diagnosis could lead to the progression of allograft dysfunction. Therefore, early intervention and amelioration of the graft dysfunction could be achieved by early prediction of AR, ATN, CNI toxicity, CAAR, and IFTA with the help of this novel approach of urinary proteome analysis.

## Figures and Tables

**Table 1:** Classification of urinary proteomes based on their molecular weight characteristics.

	Urinary proteome	Molecular weight (Da)	Sources	Examples	Graft abnormalities
1	Smaller molecular weight	1000 to 20,000	Inflammatory and interstitial cell product.	Interleukins, granzyme, perforin, and so forth.	AR, ATN, UTI, CNI toxicity, CAAR, and IFTA.

2	Low molecular weight	10,000 to 33,000	Tubular overflow.	Alpha-1 *μ*-globulin, Beta-2 *μ*-globulin, RBP, and so forth.	Physiological, UTI, and ATN.

3	Middle and high molecular weight	60,000 to 150,000	Glomerular leak.	Albumin, transferrin, and immuno-globulins.	GN, TG, and mTOR induced.

Da: Dalton, AR: acute rejection, ATN: acute tubular necrosis, UTI: urinary tract infection, CNI: calcineurin inhibitor, CAAR: chronic-antibody-associated-rejection with C4d deposits, IFTA: interstitial-nephritis-tubular-atrophy, RBP: retinol binding protein, GN: glomerulonephritis, TG: transplant glomerulopathy, and mTOR: mammalian target of rapamycin.

**Table 2:** Patterns of urinary proteome in different renal allograft dysfunctions.

	Graft dysfunction	Nature of proteomes	Sources	Urinary proteome patterns (molecular weight (Da))
1	Normal native kidney	Spill over proteomes.	Renal cells.	Peak at 9754. Increased frequency at 1668, 1817, 1687, 1782, 1882, and 2154.

2	Normal renal allograft	Beta-2 *μ*-globulin, human defensin, and so forth.	Passenger cells and spill over proteomes.	Enhancement at 3370, 3441, 3385, 4303, 4309, 4449, 5090, 4139, 5627, 5563, 5459, 10350, and 11732 (beta-2 *μ*-globulin).

3	Acute graft rejection	Adhesion molecules, cytokines, perforin, granzyme, and activation of complement cascade and coagulation cascade peptides and their degradation products.	APC, B and T-lymphocytes, NK cells, macrophages, and so forth.	Enhancement at 2003, 2807, 4756, 5872, 6990, and 19018, 25665.

4	Acute tubular necrosis	NGAL, KIM-1, and NaHE-3.	Tubular epithelial cells.	Enhancement at 5500–7500 range and at 6400, 28500, 33000, 4300, and 66000.

5	UTI			2327, 5065, 3485, 16356, 4825, and 6647.

6	CNI toxicity	Downregulation of extracellular matrix/cell adhesion components and the upregulation of secreted cyclophilin A and cyclophilin B, macrophage inhibition factor and phosphatidyl-ethanolamine-binding protein-1, and so forth.		

7	CAAR	Clusters in MS regions, which are not seen in healthy urine protein profile.	EMT cells and Lymphocytes.	2628 to 2922, 4307 to 4799, and 8303 to 8850.

8	IFTA		EMT cells and Fibroblasts.	Significant underexpressed features in the range of 2850 to 3050 from that of CAAR.

Da: Dalton, CNI: calcineurin inhibitor, CAAR: chronic antibody associated rejection with C4d deposits, IFTA: interstitial nephritis tubular atrophy NGAL: neutrophil-gelatinase-associated-ligand, KIM-1: kidney-injury-molecule-1, NaHE-3: sodium-transporter-at-apical-membrane, APC: antigen-presenting cell, MS: mass spectrometry, and EMT: epithelial-to-mesenchymal transformation.

**Table 3:** Salient features of techniques for urinary proteome analysis.

	Basic types	Specific techniques	Proteome types identified	Specific proteome identification probe/methods	Advantages	Disadvantages
1	Gel-based: electrophoresis on paper strip.	(a) Isoelectric focusing, (b) SDS-PAGE, (c) 2D-DIGE,	Proteins of 10 kDa and above	Western blotting and immune-blotting with specific antibodies against specific proteins.	Qualitative separation of proteins into low, middle, and high molecular weight protein (10 kDa and above)	Not often reproducible, quantitative assessment is difficult.

2	Gel-free: liquid chromatography, column/capillary electrophoresis with protein chips.	(a) MALDI-MS-TOF, (b) SALDI-MS-TOF, (c) iTRAQ	Peptides and small protein chains (less than 20 kDa)	MS with identification of *m*/*z* pattern.	High throughput. Multiple peptides can be assessed according to *m*/*z* chart with possible identification with known molecular weight.	Precise qualitative and quantitative isolation of individual peptide are not possible.

SDS-PAGE: sodium dodecyl sulfate polyacrylamide gel electrophoresis, 2D-DIGE: two-dimensional difference gel electrophoresis, MALDI: matrix assisted laser desorption-ionization, SELDI: surface enhanced laser desorption-ionization, MS: mass spectrometry, TOF: time-of-flight, iTRAQ: isobaric tags for relative and absolute quantification, kDa: kilo Dalton, and *m*/*z*: molecular size and charge characteristics.
